# Early costs and complications of first-line low-grade glioma treatment using a large national database: Limitations and future perspectives

**DOI:** 10.3389/fsurg.2023.1001741

**Published:** 2023-02-03

**Authors:** Kyle Tuohy, Djibril M. Ba, Debarati Bhanja, Douglas Leslie, Guodong Liu, Alireza Mansouri

**Affiliations:** ^1^ Department of Neurosurgery, Penn State Hershey Medical Center, Hershey, PA, United States; ^2^ Department of Public Health Sciences, Penn State University, University park, PA, United States; ^3^ Center for Applied Studies in Health Economics (CASHE), Penn State College of Medicine, Hershey, PA, United States; ^4^ Penn State Cancer Institute, Penn State Hershey Medical Center, Hershey, PA, United States

**Keywords:** low-grade glioma (WHO grade II), cost, resection, stereotactic biopsy, adverse events—complications, MarketScan database

## Abstract

**Introduction:**

Diffuse Low-grade gliomas (DLGG, WHO Grade II) are a heterogenous group of tumors comprising 13–16% of glial tumors. While maximal safe resection is endorsed as the best approach to DLGG, compared to more conservative interventions like stereotactic biopsy, the added costs and risks have not been systematically evaluated. The purpose of this study was to better understand the complication rates and costs associated with each intervention.

**Methods:**

A retrospective cohort study using data from the IBM Watson Health MarketScan® Commercial Claims and Encounters database was conducted, using the *International Classification of Diseases, Ninth Revision* (*ICD-9*) codes corresponding to DLGG (2005–2014). Current Procedure Terminology, 4th Edition (CPT-4) codes were used to differentiate resection and biopsy cohorts. Inverse weighting by the propensity score was used to balance baseline potential confounders (age, sex, pre-op seizure, geographic region, year, Charleston Comorbidity Index). Complication rates, hospital mortality, readmission, and costs were compared between groups.

**Results:**

We identified 5,784 and 3,635 patients undergoing resection and biopsy, respectively, for initial DLGG management. Resection was associated with greater 30-day complications (29.17% vs. 26.34%; *p* < 0.05). However, this association became non-significant after inverse propensity weighting (adjusted odds ratio = 1.09; 0.98–1.20). There was no statistically significant difference in unadjusted, 30-day hospital mortality (*p* = 0.06) or re-admission (*p* = 0.52). Resection was associated with higher 90-day total costs (*p* < 0.0001) and drug costs (*p* < 0.0001). Biopsy was associated with greater index procedure costs (*p* < 0.0001). Long-term outcomes and evaluation of DLGG subtypes was not possible given limitations in the metrics recorded in MarketScan and lack of specificity in the ICD coding system.

**Conclusion:**

Resection was not associated with an increase in the adjusted complication rate after balancing for baseline prognostic factors. Total costs and drug costs were higher with resection of DLGG, but the index procedure costs were higher for biopsy. This data should help to facilitate prospective health economic analyses in the future to understand the cost-effectiveness, and impact on quality of life, for DLGG interventions. However, the use of large national databases for studying long-term outcomes in DLGG management should be discouraged until there is greater specificity in the ICD coding system for DLGG subtypes.

## Introduction

Diffuse low-grade gliomas (DLGGs, WHO Grade II gliomas) are a heterogeneous group of tumors that comprise 13%–16% of glial tumors (1). DLGGs were traditionally considered benign and given that patients often present at a young age with minimal neurological deficit, surgeons historically opted for either clinical observation with serial imaging or stereotactic biopsy with or without adjuvant therapy (2). However, it is now known that these tumors are marked by progressive growth (3, 4) and malignant transformation in most cases (5–8).

Stereotactic biopsy was a more conservative approach that could obtain tissue for a histological and molecular diagnosis while minimizing the potential complications associated with upfront resection. Today, there is sufficient evidence to support maximal safe resection (MSR) as the best initial approach when feasible, to delay malignant progression and improve overall survival (OS) (9–12), independent of molecular markers (13, 14). However, these recommendations are based on Class III and IV evidence (15) and although the most recent European Association of Neuro-Oncology guidelines endorse this strategy (16), there are no randomized control trials (RCT) directly comparing the clinical efficacy and safety of biopsy and resection (17).

Furthermore, it is important to also evaluate the health economic impact of each procedure as well. The survival benefit with resection may be associated with a greater risk of neurological sequelae (18) and added operative costs for the patient, both of which will negatively impact a patient's quality of life post-operatively (19). Few studies have examined these effects for this procedure in the immediate post-operative period (20). In the present study, we leverage a large, national private insurance claims database in the United States (US) to achieve the following objectives: (1) compare morbidity and mortality in the early post-operative period; and (2) compare the procedural and overall costs associated with each surgical approach.

## Materials and methods

Eligible patients were identified from the IBM Watson Health MarketScan® Commercial Claims and Encounters database from January 2005 to December 2014. Our date cut-off was selected based on the transition from the International Classification of Diseases, Ninth Revision, Clinical Modification (ICD-9) to the 10th revision, which occurred in 2015. This avoids potentially confounding our data by including two different iterations of the classification system. The MarketScan database contains all claims, paid and adjudicated, at an individual-level for those enrolled in employer-sponsored health plans (21). This database has been shown to be generally representative of commercially insured patients when compared with the Medical Expenditure Panel Survey (22). For regional comparisons, the States encompassed within each region are based on the United States Census Regions and Divisions (Supplementary Figure S1).

This study was approved by the institutional review board at the Penn State Milton S. Hershey Medical Center. Informed consent was not required since all data was deidentified prior to access.

### Subject eligibility

**Figure 1:**
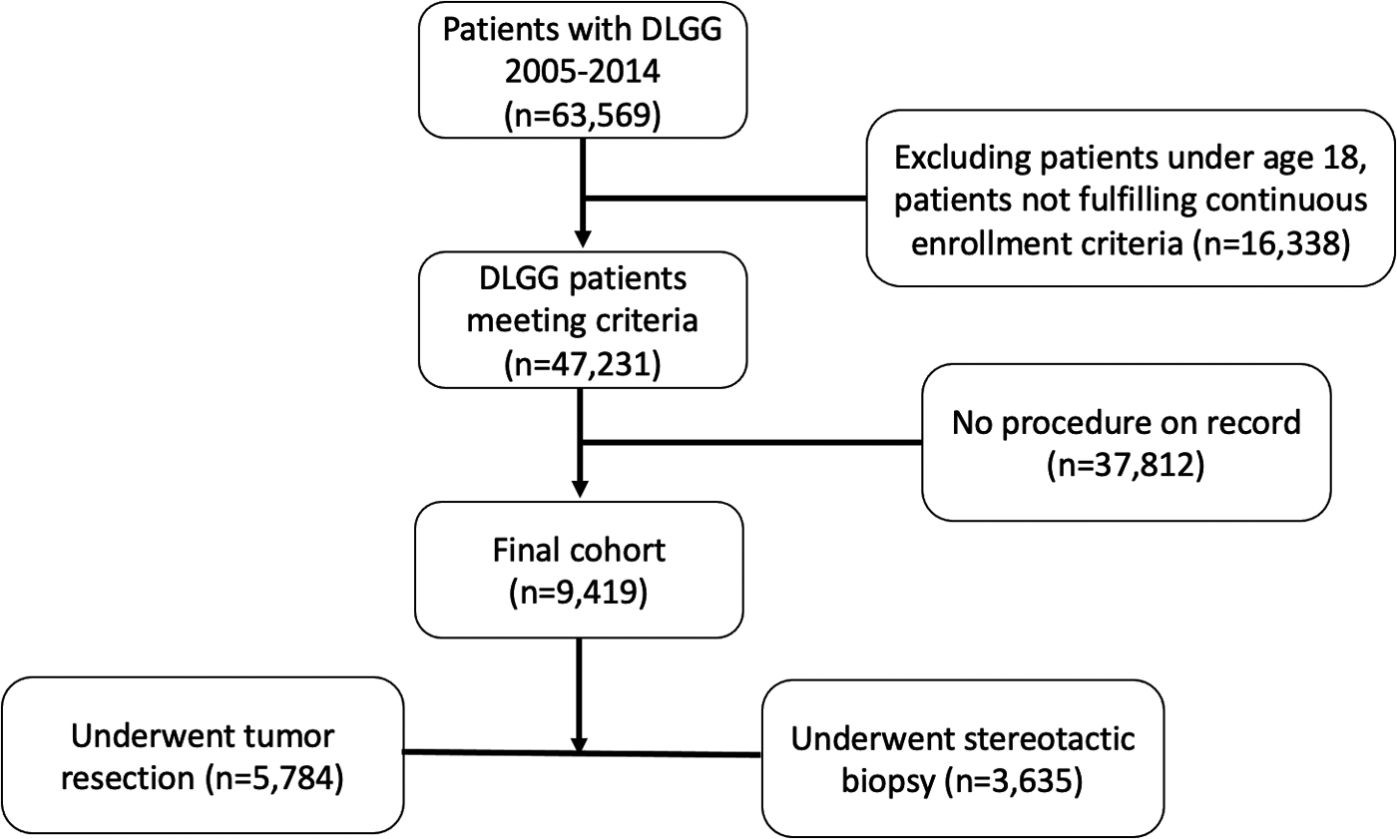
Flow chart for case identification and exclusion.

Patients over the age of 18 years undergoing stereotactic biopsy or resection for a DLGG were included in this analysis (Figure 1). A diagnosis of DLGG was determined using the ICD-9 code of 225.0. Of these patients, we identified those undergoing resection or stereotactic biopsy using Current Procedural Terminology, 4th Edition (CPT-4) codes corresponding to each procedure. The full list of codes used in this study are found in the Supplementary Table S1. Patients were only included if they had at least 6 months of pre-diagnosis index dates of enrollment and at least 3 months of post-index dates of enrollment to allow for adequate time to evaluate for adverse events and costs.

The Charleston Comorbidity Index (CCI) was used to identify the presence and severity of comorbid conditions as a marker for complication risk at baseline. This variable was chosen because previous MarketScan database studies have validated its use. Furthermore, other brain tumor specific indices such as the Pignatti (23) and Sawaya (24) scores could not be used since the variables that go into these scores cannot be obtained from the MarketScan database.

### Outcome measures

Adverse events potentially attributable to the surgical intervention or post-operative course were identified with ICD-9 codes (Supplementary Table 1). All adverse events were evaluated during the 30-day period after the index procedure. To avoid attributing preexisting conditions as surgery-related complications, conditions were only considered new if the diagnostic code was not present during the 6 months prior to surgery. Mortality and re-admission to the hospital were also recorded within this period.

Regarding costs, the total payments made by the individual or a third-party payer were used. All costs were adjusted to 2014 dollars using the United State Department of Lar Medical Care Consumer Price Index (25). A list of drugs used to evaluate chemotherapy and anti-epileptic drug costs can be found in the Supplementary Table S2.

### Statistical analysis

Raw data are presented using descriptive statistics. Continuous variables are presented using mean and standard deviation. Dichotomous data are presented as frequencies and percentages. Univariate analysis was performed to assess statistical significance of differences in proportions using a Chi-Square test for categorical variables and t-test for continuous variables.

The level of accepting statistical significance was set at 0.05, except for the individual complications, which were set to 0.004. We chose to adjust for multiple variables using the Bonferroni correction in this case to account for the analysis of 14 dependent variables (0.05/14 = 0.004). Multivariable analysis was conducted using logistic regression to assess the association between procedures (resection & biopsy) and complications, mortality, and readmission, respectively. Stereotactic biopsy was used as the reference when calculating adjusted odds ratios (OR).

To further test the robustness of our results, we conducted a sensitivity analysis inverse weighted by the propensity score (26) to balance baseline data between participants in each group. Propensity scores were derived from predicted probabilities of being in each surgery group estimated in logistic regression models that contained covariates that may have acted as confounding variables (age, sex, pre-operative seizures, geographic region, year of operation, and CCI stratified into 0, 1, or ≥2).

## Results

### Patient characteristics

We identified 5,784 patients undergoing resection and 3,635 undergoing biopsy for initial DLGG management (Table 1). Baseline characteristics were significantly different between groups. Female patients represented 50.24% of the resection group and 60.19% of the biopsy group (*p* < 0.0001 for both). The mean (SD) age was 46.0 (12.9) and 47.8 (11.5) years for resection and biopsy groups, respectively (*p* < 0.0001). The biopsy group had more patients in all age ranges except the youngest group (18–34 years). There were significantly more patients in the resection group that had seizures prior to their operation (32.35% vs. 23.03%; *p* < 0.0001). Lastly, more patients in the resection group had CCI scores of 0 (26.04% vs. 37.80%) or 1 (7.87% vs. 13.67%), whereas the biopsy group had more patients with scores greater than or equal to 2 (66.10% vs. 48.53%) (*p* < 0.0001 for all).

**Table 1:** Low-Grade Glioma Patient Characteristics in Total Cohort, Stratified by Procedure (2005–2014).

Patient Characteristics		Supratentorial Resection *N* = 5,784	Stereotactic Biopsy *N* = 3,635	*p*-Value
Sex, *n* (%)	Female	2,906 (50.24)	2,188 (60.19)	<0.0001
Age, *n* (%)	Mean (SD)	46.0 (12.9)	47.8 (11.5)	<0.0001
	18–34	1,230 (21.27)	528 (14.53)	<0.0001
	35–44	1,080 (18.67)	704 (19.37)	<0.0001
	45–54	1,606 (27.77)	1,146 (31.53)	<0.0001
	55–64	1,868 (32.30)	1,257 (34.58)	<0.0001
Geographic Region, *n* (%)	Northeast	1,021 (17.63)	806 (22.17)	<0.0001
	North Central	1,288 (22.27)	761 (20.94)	<0.0001
	South	2,477 (42.83)	1,375 (37.83)	<0.0001
	West	919 (15.89)	628 (17.28)	<0.0001
	Unknown	79 (1.37)	65 (1.79)	<0.0001
Pre-Operative Seizures, *n* (%)	Yes	1,871 (32.35)	837 (23.03)	<0.0001
Charlson Comorbidity Index, *n* (%)	0	1,506 (26.04)	1,374 (37.80)	<0.0001
	1	455 (7.87)	497 (13.67)	<0.0001
	≥2	3,823 (66.10)	1,764 (48.53)	<0.0001

### Adverse events

After excluding those with a complication code prior to surgery, there were 4,094 total patients remaining, of which 1,144 (27.94%) had a complication code post-operatively. Resection was associated with a higher rate of overall complications compared to biopsy (29.17% vs. 26.34%; *p* = 0.0456) (Table 2). However, there was no significant difference in overall complications after conducting a sensitivity analysis based on inverse propensity weighting (adjusted OR = 1.088; CI = 0.984–1.204; *p* = 0.10). There was no significant difference in 30-day re-admission rates (13.07% vs. 13.54%; *p* = 0.5174) or hospital mortality (0.21% vs. 0.06%; *p* = 0.0615). The rates of each individual complication are shown in Table 3. There were no statistically significant differences between the resection and biopsy groups for any individual complication.

**Table 2:** Rates of postoperative complications associated with resection and biopsy.

	Supratentorial Resection *n* (%)	Stereotactic Biopsy *n* (%)	Odds Ratio (95% CI)	*p*-Value
Unadjusted total complications within 30 days post-op	677 (29.17)	467 (26.34)	1.15 (1.00–1.32)	0.0456*
Adjusted total complications by propensity weighting	–	–	1.09 (0.98–1.20)	0.10
Adjusted readmission within 30 days post-op	756 (13.07)	492 (13.54)	0.91 (0.80–1.04)	0.52
Mortality within 30 days post-op	12 (0.21)	2 (0.06%)	–	0.06

*Statistically significant.

**Table 3:** Frequency of individual complications associated with maximal safe resection and stereotactic biopsy procedures.

Type of Complication	Supratentorial Resection, *n* (%)	Stereotactic Biopsy, *n* (%)	Adjusted Odds Ratio (95% CI)	*p*-Value
Delirium	19 (0.82)	7 (0.39)	1.916 (0.758–4.846)	0.09
Deep Vein Thrombosis	80 (3.45)	46 (2.59)	1.394 (0.930–2.089)	0.12
Dysphagia/ Dystonia	53 (2.28)	44 (2.48)	0.842 (0.540–1.314)	0.68
Dysrhythmia	73 (3.15)	66 (3.72)	0.856 (0.589–1.244)	0.31
General Neurological Complication	253 (10.90)	199 (11.22)	1.010 (0.813–1.255)	0.74
General Neurosurgical Complication	67 (2.89)	45 (2.54)	1.278 (0.840–1.945)	0.50
Hematoma/ Hemorrhage	59 (2.54)	40 (2.26)	1.089 (0.698–1.702)	0.56
Pulmonary Embolism	25 (1.08)	14 (0.79)	1.596 (0.783–3.256)	0.35
Other Cardiac Complication	0 (0.00)	1 (0.06)	–	0.43
Respiratory Complication	113 (4.87)	77 (4.34)	1.010 (0.729–1.400)	0.43
Wound Infection	77 (3.32)	35 (1.97)	1.668 (1.073–2.595)	0.009
Vascular Injury	12 (0.52)	9 (0.51)	1.357 (0.527–3.492)	0.97
Seizure	303 (7.74)	211 (7.54)	–	0.58
Myocardial Infarction	8 (0.14)	4 (0.11)	–	0.71

### Costs

Cost data are summarized in Table 4. Resection was associated with higher total 90-day costs ($56,093.34 vs. $43,219.44; *p* < 0.0001) and 90-day drug costs ($4,005.28 vs. $2,277.44; *p* < 0.0001). Similarly, resection was also associated with higher out-of-pocket expenses for total 90-day costs ($1,164.13 vs. $811.03) and 90-day drug costs ($210.84 vs. $153.61) (*p* < 0.0001 for both). Biopsy was associated with higher index procedure costs ($39,043.06 vs. $40,661.28; *p* = 0.001). However, there was no difference between groups for out-of-pocket expenses for the index procedure ($1,054.79 vs. $1,077.02; *p* = 0.53).

**Table 4:** Costs associated with resection and stereotactic biopsy in overall cohort (United States dollars).

Costs	Supratentorial Resection		Stereotactic Biopsy		*p*-Value
	Mean	Standard Deviation	Mean	Standard Deviation	
Total Costs within 90 days Post-Op	56,093.34	67,974.42	43,219.44	65,462.92	<0.0001*
Costs within 90 days Post-Op, Out-of-Pocket	1,164.13	4,857.70	811.03	1471.07	<0.0001*
Total Drug Costs within 90 days Post-Op	4,005.28	7,177.83	2,277.44	5,543.41	<0.0001*
Drug Costs within 90 days Post-Op, Out-of-Pocket	210.84	472.01	153.61	376.56	<0.0001*
Total Index Procedures Costs	39,043.06	44,390.94	40,661.28	47,068.13	0.001*
Index Procedures Costs, Out-of-Pocket	1,054.79	2,671.90	1,077.02	2,319.64	0.53

*Statistically significant.

Lastly, we evaluated chemotherapy and anti-epileptic drug costs specifically (Table 5). We found no difference in cost between groups for chemotherapy drug use ($12,716.95 vs. $12,751.83; *p* = 0.97). For anti-epileptic drugs, the resection group had significantly greater costs compared to biopsy ($376.48 vs. $253.19; *p* < 0.0001). These relationships remained the same when comparing out-of-pocket expenses alone.

**Table 5:** Seizure and chemotherapy drug costs, 90-days post-op.

Costs	Supratentorial Resection		Stereotactic Biopsy		*p*-Value
	Mean	Standard Deviation	Mean	Standard Deviation	
Total chemotherapy costs	12,716.95	6782.46	12,751.83	6976.36	0.97
Chemotherapy costs, out-of-pocket	301.14	673.68	274.41	378.99	0.69
Total anti-epileptic drug costs	376.48	658.87	253.19	614.56	<0.0001*
Anti-epileptic drug costs, out-of-pocket	55.98	108.64	40.30	74.35	<0.0001*

*Statistically significant.

## Discussion

There is now a wealth of evidence to support maximal safe resection as the best initial strategy for improving survival and delaying malignant progression (15). As we push toward making DLGG a chronic disorder, however, it is imperative to better understand costs and complications associated with each approach, to fully appreciate cost-effectiveness and effects on quality of life. Using the MarketScan database, we report nation-wide data demonstrating the immediate adverse events and costs associated with resection and biopsy for DLGG management.

Groups were balanced by comorbid conditions through our propensity score analysis, as well as geographic region and year in order to control for potential temporal or geographical influences on surgical practice patterns (27). Upon balancing for these factors using propensity analysis, we found no difference in overall complication rates between treatment groups. This suggests that the greater risk for complications with resection may not be due to the procedure itself, but the elevated baseline risk of the patient population undergoing this intervention given that complications may occur in up to 20%–25% of patients treated with resection, compared to 3%–6% of those with biopsy (18, 28). This is further supported by the fact that we did not find a difference in risk for any of the individual complications as well.

There was also no associated increase in re-admission rates or differences in mortality. Mortality has been considered low for both resection and biopsy, but can be estimated to be around 1.5% (29) and 0.9% (30), respectively. While a 26% morbidity rate in our biopsy cohort is higher than expected, it may be necessary for the neurosurgery community to more comprehensively evaluate complication rates using data derived from large national registries. Additionally, it is important to emphasize that the severity of these complications is not captured by the MarketScan database.

Although short-term outcomes were sufficient to evaluate the immediate post-operative complications associated with each procedures, we were unable to collect long-term outcomes as part any secondary analyses. Theoretically, these could be evaluated, but we felt this data would be unreliable given how MarketScan collects this information. MarketScan will only collect data on those who possess private health insurance, and this data is collected *via* codes submitted by healthcare providers. Therefore, patients who die at home may not have their mortality data collected, nor would those who lost private insurance, such as those who became uninsured or transitioned to Medicare/Medicaid without supplemental private insurance.

Regarding cost, we found that resection had higher 90-day total and drug costs, including the out-of-pocket values for each. This difference was not attributable to chemotherapy drug costs, as these were similar in both cohorts. However, anti-epileptic drug costs were higher in the resection cohort, which may represent increased seizure severity in this group. To assess all costs more accurately, including tangible and intangible ones, prospective health economic analyses that take into consideration the patient perspective with appropriate time horizons are necessary.

The overall index procedure costs were higher for biopsy, but the difference in out-of-pocket expenses for the index procedure was not statistically significant between groups. These are the costs incurred on the same date as the initial resection or biopsy procedure itself. Further research is needed into why these costs were higher for biopsy when no others were. It may have to do with facility costs or other administrative costs that are higher for biopsy, such as charges that do not end up being seen by the patient given that index procedure cost was not significantly different from resection when looking at “out-of-pocket” costs alone. Biopsy procedures likely have more disposable medical equipment involved. In addition, stereotactic biopsy equipment is expensive, and this may be transmitted into higher cost to the healthcare system on the day of the procedure, which is absorbed by the hospital and not seen by the patient. Given that this cost does not include post-operative care, it is not altered by post-operative complications, which may have contributed to the higher 90-day costs for resection. Biopsy is a more cost-effective procedure in the treatment of many tumors (31), but our results suggest that the cost benefit for DLGG may be more so due to the post-operative course than the index procedure itself.

### Limitations and future perspectives

There is a scarcity of research regarding cost-effectiveness for DLGG management (32). Due to the relatively low prevalence of DLGG and their heterogeneity, prospective studies may prove challenging. Large-scale registries of prospective observational data may alleviate some of these challenges by allowing for large scale analysis more efficiently than true prospective trials or RCTs (33). However, the benefits of this approach must be weighed against the expected limitations.

Some of these limitations are inherent to any study using an administrative claims database. Given the retrospective nature of these analyses, we are limited to the predetermined variables collected by the database and the information defined by each clinical code. Regarding our findings, we were unable to include all known or potential prognostic markers related to DLGG management, which play a role in the decision-making process when determining the best surgical approach. In addition, we were unable to assess the severity of each complication. Our cohort included only those patients with commercial health insurance and therefore these results may not be generalizable to all patient populations, but it should be noted that the MarketScan population is representative of the largest segment of American healthcare users.

However, we also recognized specific limitations in using large administrative databases for studying DLGG. First, long-term outcomes were not able to be assessed. Although there was sufficient data to evaluate our objectives in the immediate post-operative period, it prohibits the use of these databases for extended outcomes that are important for slowly progressive diseases like DLGG. For example, many patients will be treated with chemotherapy longer than the 3 months analyzed here and although we may be able to extrapolate our results to the full treatment duration, it would be ideal to expand the length of data collection to capture all chemotherapy cycles.

Classification of disease is done *via* the ICD coding system, which is an imperfect method for properly classifying intracranial tumors. First, this code encompasses also encompasses benign tumors such as grade I gliomas, ependymomas, choroid plexus papillomas, and dermoid/epidermoid cysts. We believe that we excluded many of these entities by only including supratentorial lesions in patients over the age of 18, and by the fact that these are much less common than DLGG overall (1). They likely made up only a very small number in our cohort, but it nevertheless hinders the specificity of our methodology. More importantly, however, it makes it nearly impossible to perform such studies on these types of lesion given the low prevalence of each. Second, the ICD system lacks the specificity to differentiate between subtypes of DLGG. This includes no differentiation based on molecular features, which are now featured heavily in diagnosis (34), as well as tumor location. These factors affect prognosis and treatment decisions, and the lack of these variables greatly hinders a clinician's ability to apply morbidity and cost information to a specific patient.

In addition, The CPT coding system provides its own limitations when it comes to evaluating surgical resection. These codes do not specify the extent of resection (ex. gross total vs. sub-total), which is important given the known benefit of MSR for these patients (5, 11, 15). This difference is unlikely to affect index procedure costs, but may play a role in 90-day total and drug costs. It may also play a role in post-operative complications, as a more aggressive surgery like gross total resection may be associated with a greater risk for complications. Any such risk will likely be outweighed by the well-established benefit to survival and progression, however it is still important to understand this risk when discussing these procedures with patients.

Given these limitations, we recommend against the use of large administrative databases to continue studying outcomes in DLGG management until there is greater specificity in the ICD coding system. Ideally, such studies should be conducted on prospectively collected databases designed specifically for DLGG, in which appropriate variables can be collected that are relevant to this patient population. Prospective health economic analyses, incorporating both procedural costs and concordant impact on quality of life, would help delineate the full benefit of MSR.

We acknowledge that the goal of resection and biopsy are not the same. Biopsy is performed as a diagnostic procedure, often to guide future treatment decisions, whereas resection is an evidence-based therapeutic treatment. This study is not meant to question the utility of resection, and without outcomes data it would be impractical to do so. However, we felt that it would be impractical to present costs and complications without a comparison group. Biopsy was chosen as a comparator procedure since this was historically considered a viable alternative to resection. We hope that this information may serve as a basis for evaluating the cost-effectiveness of DLGG management in the future through prospective health economic analyses.

In general, there is a scarcity of cost-effectiveness research in the neurosurgical literature (35). These analyses require adequate cost data and quantitative, clinically relevant unit of health like mortality or life-years gained. From this, an incremental cost-effectiveness ratio can be obtained, in which the extra cost per extra unit of health gained can be compared between two interventions. The preferred unit of health for such analyses is the quality-adjusted life year (36), which in addition to knowing the absolute number of years gained by an intervention, it also requires an understanding of potential adverse events that may affect quality of life. This should include adverse effects on mental health (37), which are often not considered. This underscores the need for further research that can further elicit this important prerequisite information.

## Conclusion

Few studies on DLGG management have evaluated national complications and costs, both of which impact a patient's quality of life post-operatively. To our knowledge, the present study is the first to employ the MarketScan research database in the DLGG population for this purpose. This data can be used in future health economic analyses to better understand the cost-effectiveness of DLGG interventions. However, our experience has also highlighted significant limitations in the utilization of administrative databases for the study of DLGG. Long-term outcomes should instead be assessed *via* prospectively collected databases designed specifically for DLGG, at least until improvements are made in general database variables and ICD coding.

## Data Availability

The original contributions presented in the study are included in the article/**Supplementary Material**, further inquiries can be directed to the corresponding author/s.
