# Supplementary material for: Early costs and complications of first-line low-grade glioma treatment using a large national database: Limitations and future perspectives
Source: Front Surg. 2023 Feb 3;10:1001741. doi: 10.3389/fsurg.2023.1001741 (PMC9935584; doi:10.3389/fsurg.2023.1001741)
Supplement: Supplementary file 1 [file Table1.docx]

| **Diagnosis Codes** | |
| --- | --- |
| **Descriptor** | **ICD-9 Code** |
| Benign neoplasm of the brain, unspecified | 225.0 |
| **Procedure Codes** | |
| **Descriptor** | **CPT Code** |
| Stereotactic Biopsy | 61750, 61751, 61781 |
| Supratentorial Resection | 61510, 61518, 61305, 61304 |
| **Complication Codes** | |
| **Descriptor** | **Number** |
| Acute myocardial infarction (ICD-9) | 410.0-410.9 |
| Delirium (ICD-9) | 293.1-293.9 |
| Deep vein thrombosis (ICD-9) | 453.4, 451, 453.1, 453.2, 453.8, 453.9 |
| Dysphagia/dystonia (ICD-9) | 478.3, 784.4, 787.2 |
| General neurosurgical complication (ICD-9) | 997.0 |
| General neurological complication (ICD-9) | 430-436, 438.2, 438.3, 438.4, 438.5 |
| Hematoma/hemorrhage (ICD-9) | 998.1 |
| Other cardiac complication (ICD-9) | 997.1 (NOS), 998.0 (post-op shock, NOS) |
| Pulmonary embolism (ICD-9) | 415.11, 415.19 |
| Respiratory complication (ICD-9) | 997.3, 512.1, 518.4, 518.5, 518.7, 507.0, 518.8 |
| Seizure (ICD-9) | 345, 780.3 |
| Urinary tract infection (ICD-9) | 996.64 |
| Vascular injury (ICD-9) | 900.0, 900.82, 900.89, 900.9, 997.02, 954.0 |
| Wound complication (ICD-9) | 998.31, 998.32, 998.81, 998.83, 998.5, 999.3 |
| Post-op infection (CPT) | 10140, 10160, 10180, 12020, 12021, 20005, 21501, 22010 |

ICD-9, *International Classification of Diseases, Ninth Revision*; CPT, Current Procedure Terminology, 4^th^ Edition Codes.
