# Supplementary material for: Early costs and complications of first-line low-grade glioma treatment using a large national database: Limitations and future perspectives
Source: Front Surg. 2023 Feb 3;10:1001741. doi: 10.3389/fsurg.2023.1001741 (PMC9935584; doi:10.3389/fsurg.2023.1001741)
Supplement: Supplementary file 2 [file Table2.docx]

| **Chemotherapy drugs** | **Anti-epileptic drugs** |
| --- | --- |
| Temozolomide, Procarbazine, Lomustine, Vincristine | Brivaracetam, Clobazam, Felbamate, Lamotrigine, Levetiracetam, Perampanel, Rufinamide, Topiramate, Valproate, Zonisamide, Carbamazepine, Cenobamate, Eslicarbazepine, Gabapentin, Lacosamide, Oxcarbazepine, Phenobarbital, Phenytoin, Pregabalin, Primidone, Stiripentol, Tiagabine, Vigabatrin |
