# Supplementary material for: Early costs and complications of first-line low-grade glioma treatment using a large national database: Limitations and future perspectives
Source: Front Surg. 2023 Feb 3;10:1001741. doi: 10.3389/fsurg.2023.1001741 (PMC9935584; doi:10.3389/fsurg.2023.1001741)
Supplement: Supplementary file 3 [file Datasheet1.pdf]

Census Regions and Divisions of the United States

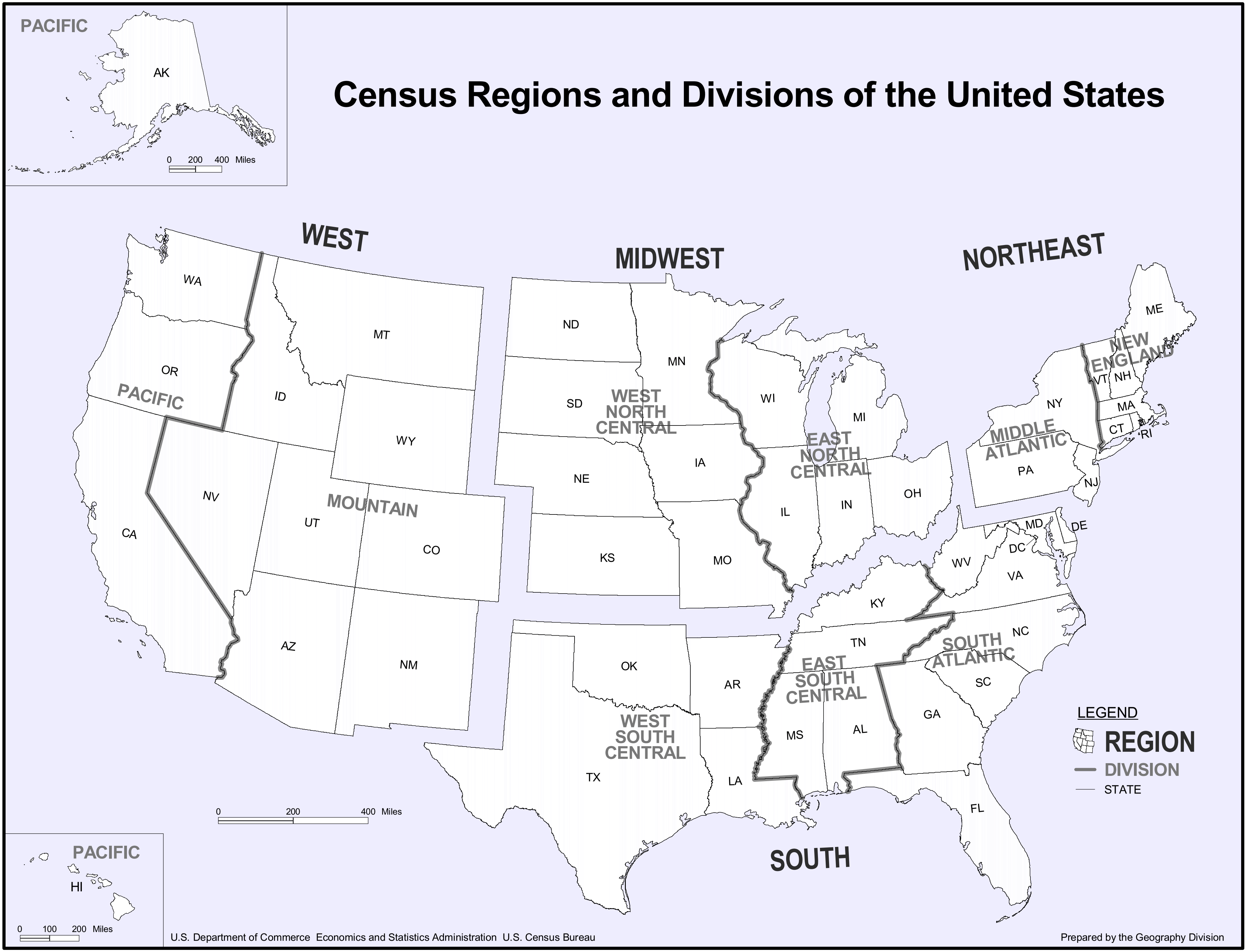

U.S. Department of Commerce Economics and Statistics Administration U.S. Census Bureau

Prepared by the Geography Division

Census Bureau Regions and Divisions with State FIPS Codes

Region I: Northeast

Division I: New England

Connecticut (09)
Maine (23)
Massachusetts (25)
New Hampshire (33)
Rhode Island (44)
Vermont (50)

Division 2: Middle Atlantic

New Jersey (34)
New York (36)
Pennsylvania (42)

Region 2: Midwest*

Division 3: East North Central

Indiana (18)
Illinois (17)
Michigan (26)
Ohio (39)
Wisconsin (55)

Division 4: West North Central

Iowa (19)
Kansas (20)
Minnesota (27)
Missouri (29)
Nebraska (31)
North Dakota (38)
South Dakota (46)

Region 3: South

Division 5: South Atlantic

Delaware (10)
District of Columbia (11)
Florida (12)
Georgia (13)
Maryland (24)
North Carolina (37)
South Carolina (45)
Virginia (51)
West Virginia (54)

Division 6: East South Central

Alabama (01)
Kentucky (21)
Mississippi (28)
Tennessee (47)

Division 7: West South Central

Arkansas (05)
Louisiana (22)
Oklahoma (40)
Texas (48)

Region 4: West

Division 8: Mountain

Arizona (04)
Colorado (08)
Idaho (16)
New Mexico (35)
Montana (30)
Utah (49)
Nevada (32)
Wyoming (56)

Division 9: Pacific

Alaska (02)
California (06)
Hawaii (15)
Oregon (41)
Washington (53)

**Prior to June 1984, the Midwest Region was designated as the North Central Region.*
